# The effect of active video games by ethnicity, sex and fitness: subgroup analysis from a randomised controlled trial

**DOI:** 10.1186/1479-5868-11-46

**Published:** 2014-04-03

**Authors:** Louise Foley, Yannan Jiang, Cliona Ni Mhurchu, Andrew Jull, Harry Prapavessis, Anthony Rodgers, Ralph Maddison

**Affiliations:** 1 National Institute for Health Innovation, School of Population Health, University of Auckland, Private Bag 92019, Auckland 1142, New Zealand; 2 School of Nursing, University of Auckland, Private Bag 92019, Auckland 1142, New Zealand; 3 School of Kinesiology, University of Western Ontario, London, ON N6A 5B9, Canada; 4 The George Institute for Global Health, PO Box M201, Missenden Road, Sydney, NSW 2050, Australia

**Keywords:** Active video games, Overweight and obesity, Subgroup analysis

## Abstract

**Background:**

The prevention and treatment of childhood obesity is a key public health challenge. However, certain groups within populations have markedly different risk profiles for obesity and related health behaviours. Well-designed subgroup analysis can identify potential differential effects of obesity interventions, which may be important for reducing health inequalities. The study aim was to evaluate the consistency of the effects of active video games across important subgroups in a randomised controlled trial (RCT).

**Findings:**

A two-arm, parallel RCT was conducted in overweight or obese children (n = 322; aged 10–14 years) to determine the effect of active video games on body composition. Statistically significant overall treatment effects favouring the intervention group were found for body mass index, body mass index z-score and percentage body fat at 24 weeks. For these outcomes, pre-specified subgroup analyses were conducted among important baseline demographic (ethnicity, sex) and prognostic (cardiovascular fitness) groups. No statistically significant interaction effects were found between the treatment and subgroup terms in the main regression model (p = 0.36 to 0.93), indicating a consistent treatment effect across these groups.

**Conclusions:**

Preliminary evidence suggests an active video games intervention had a consistent positive effect on body composition among important subgroups. This may support the use of these games as a pragmatic public health intervention to displace sedentary behaviour with physical activity in young people.

## Findings

### Background

The prevention and treatment of childhood obesity is a key public health challenge [1,2]. Within populations, subgroups have different risk profiles for obesity and related health behaviours. Marked differences exist in the prevalence of childhood overweight and obesity among minority or indigenous groups internationally [3] and in New Zealand [4], with resulting health inequalities. Additionally, evidence suggests that minority groups have greater engagement in sedentary screen-based pursuits, which have been implicated in the development of obesity [5]. Screen-time is known to differ by sex as well as ethnicity. Boys are greater users of screen-based media than girls in the United States [6], Australia [7] and New Zealand [4].

Subgroup analysis can be used to identify the consistency of main trial effects across different groups of participants. This approach is particularly important for public health interventions and may help inform policy development and implementation strategies for subgroups where known health inequalities exist [8]. However, such analysis is often criticised due to lack of sufficient statistical power with increased likelihood of Type I error [9,10]. Guidelines suggest subgroup analysis is appropriate when (a) the main trial effect is positive, (b) there is a plausible rationale for examining a particular subgroup specified *a priori*, and (c) the results of analyses are considered exploratory [8,10,11]. When used appropriately, subgroup analysis may be used to provide additional information on messaging to help tackle health inequalities [8]. Few community-based physical activity interventions have reported the main trial effects by important subgroups [12].

The Electronic Games to Aid Motivation to Exercise (eGAME) study was a randomised controlled trial (RCT) to evaluate the effect of active video games on body composition, physical activity and cardiovascular fitness in overweight and obese children in New Zealand [13]. A modest but statistically significant treatment effect favouring the intervention group on change in body mass index [BMI] (kg/m^2^ and z-score) and percentage body fat was found at 24 weeks post intervention. As the only large active video games RCT conducted to date, the study provides an opportunity to explore the consistency of main trial effects across important subgroups.

In the context of the eGAME trial, ethnicity and sex are subgroups of interest because of the different risk profiles for overweight and screen-based behaviour. Additionally, cardiovascular fitness may be important, as a recent meta-analysis indicated an inverse relationship between cardiovascular fitness and metabolic syndrome and obesity in children [14]. Therefore, the aim of this paper was to conduct a pre-specified subgroup analysis of the eGAME study to examine the consistency of trial effects by ethnicity, sex and baseline cardiovascular fitness.

## Methods

### Design

The eGAME trial was a two-arm, parallel RCT to evaluate the effect of active video games on body composition, physical activity and physical fitness in overweight and obese children. Ethical approval was granted (NTY/07/09/099), and the trial was registered (ACTRN12607000632493). Details regarding the methods [15] and main results [13] of the eGAME trial have been reported, and are briefly described below.

### Participants

A total of 322 overweight or obese [16] children aged 10–14 years, who regularly used traditional sedentary video games, were randomly allocated to a 24 week active video games intervention (n = 160) or control (n = 162). Participants were excluded if they already owned active video games or had a medical contraindication to performing physical activity.

### Procedure

Intervention participants received a home-based active video games intervention. The Sony PlayStation EyeToy was used, which employs a USB motion-capture camera to place a picture of the gamer on screen, which the gamer then interacts with. Control participants continued as normal and received the active video games at the end of the study. Assessments were conducted at baseline, 12 weeks and 24 weeks. At each time point, participants had their height and weight measured, underwent bioelectrical impedance analysis and completed a field test of cardiovascular fitness, amongst other measures [17].

### Outcomes

The primary outcome was change from baseline in BMI (kg/m^2^) and standardised BMI (z-score).

### Statistical analysis

Analyses were performed on the principle of intention-to-treat, including all randomised participants who provided at least one valid post-baseline measure. Analysis of covariance regression models were used to evaluate main treatment effects, adjusting for baseline outcome, age, sex, and ethnicity. When a significant main treatment effect was detected, further analyses were conducted to assess the consistency of the effect across subgroups: (a) self-identified ethnicity (Māori, Pacific or New Zealand European/Other); (b) sex (male or female); and (c) baseline VO_2max_ (tertiles low <23.8, medium 23.8-28.3, high >28.3 ml/kg/min). The specification of the subgroups for ethnicity and sex were defined *a priori*, however, the decision to split cardiovascular fitness into tertiles was made *post hoc* to ensure an equal distribution of numbers across the categories.

The interaction effect between the treatment and subgroup terms was tested in the main model. A significant interaction would indicate differential treatment effects among the subgroups. The same regression models were carried out for participants within individual subgroups to estimate the magnitude of the intervention effect in each subgroup. When the subgroups analyses on ethnicity and sex were conducted, the corresponding term in the adjusted model was removed.

The outcomes of interest were change from baseline in BMI, BMI z-score and percentage body fat. Statistical analyses were performed using SAS version 9.2 (SAS Institute Inc, Cary, NC) and R 2.14.0 (The R Foundation for Statistical Computing, Vienna, Austria). Statistical tests were two-sided at a 5% significance level. No adjustment on multiple comparisons was considered.

### Statistical power

As a standard two-arm RCT, the sample size calculation was powered on the primary outcome only, using all randomised participants. Therefore, sample size calculations for sub-group analyses were not conducted. *Post hoc* power calculation was not considered appropriate for multiple subgroup analyses.

## Results

Table 1 presents baseline descriptive data in each arm by subgroup. The final sample consisted of 17% Māori (indigenous ethnic group) and 26% Pacific (minority ethnic group), compared with 57% New Zealand European/Other. Furthermore, the majority of participants were males (73%).

**Table 1:** Descriptive summary of baseline outcomes by subgroup

	**Intervention**			**Control**			**Overall**		
	**n**	**Mean**	**SD**	**n**	**Mean**	**SD**	**n**	**Mean**	**SD**
**BMI (kg/m**^**2**^**)**									
Gender									
Female	44	25.96	4.25	43	26.51	4.65	87	26.23	4.43
Male	116	25.52	4.03	119	25.47	4.09	235	25.5	4.05
Ethnicity									
NZEO	92	24.73	3.63	91	24.55	3.85	183	24.64	3.73
Maori	27	26.77	4.31	28	25.87	2.96	55	26.31	3.68
Pacific	41	26.96	4.44	43	28.19	4.75	84	27.59	4.61
Baseline VO_2max_									
Low	51	27.56	4.27	52	27.89	5.01	103	27.73	4.64
Medium	59	25.81	4.1	54	25.22	4.04	113	25.53	4.06
High	49	23.49	2.68	54	24.21	2.68	103	23.87	2.69
All	160	25.64	4.08	162	25.75	4.25	322	25.7	4.16
**BMI z-score**									
Gender									
Female	44	1.11	0.97	43	1.29	0.96	87	1.2	0.97
Male	116	1.31	1.2	119	1.24	1.14	235	1.27	1.17
Ethnicity									
NZEO	92	1.5	1.12	91	1.47	1.19	183	1.49	1.15
Maori	27	1.22	0.9	28	0.93	0.54	55	1.08	0.74
Pacific	41	0.73	1.18	43	1.01	1.08	84	0.87	1.13
Baseline VO_2max_									
Low	51	1.73	1.1	52	1.83	1.26	103	1.78	1.18
Medium	59	1.25	1.17	54	1.13	1	113	1.19	1.09
High	49	0.78	0.98	54	0.79	0.74	103	0.79	0.86
All	160	1.26	1.14	162	1.25	1.1	322	1.25	1.12
**Percentage body fat (%)**									
Gender									
Female	44	34.38	4.78	43	35.16	5.73	87	34.76	5.26
Male	114	31.24	6.88	117	31.5	6.37	231	31.37	6.61
Ethnicity									
NZEO	91	32.33	6.12	90	33.61	5.95	181	32.96	6.05
Maori	27	33.67	5.63	28	31.24	6.24	55	32.43	6.02
Pacific	40	30.59	7.67	42	30.9	7.06	82	30.75	7.32
Baseline VO_2max_									
Low	49	36.21	4.5	51	36.21	5.55	100	36.21	5.04
Medium	59	32.27	5.4	54	32.53	5.74	113	32.4	5.54
High	49	27.78	6.82	53	28.9	5.52	102	28.36	6.17
All	158	32.12	6.51	160	32.48	6.39	318	32.3	6.44

BMI – body mass index; kg – kilogram; m – metre; n – number; NZEO – New Zealand European/Other; SD – standard deviation; VO_2max_ – maximal oxygen uptake.

There were no significant interactions between the treatment and subgroups in all regression models (p = 0.36 to 0.93), indicating no statistical evidence of treatment differences among these subgroups of interest (Table 2).

**Table 2:** Estimates of intervention effect in subgroups of interest

**Change from baseline outcome**	**Intervention**	**Control**	**Difference**	**Lower 95% CI**	**Upper 95% CI**	**P of interaction**
**BMI (kg/m**^**2**^**)**						
Gender						0.69
Female	0.29	0.51	−0.22	−0.68	0.24	
Male	−0.19	0.12	−0.30	−0.57	−0.04	
Ethnicity						0.46
NZEO	−0.01	0.23	−0.24	−0.51	0.03	
Maori	0.18	0.68	−0.50	−1.21	0.21	
Pacific	0.04	0.25	−0.21	−0.66	0.24	
Baseline VO_2max_						0.93
Low	−0.12	0.10	−0.22	−0.62	0.18	
Medium	0.23	0.44	−0.21	−0.62	0.21	
High	0.03	0.36	−0.34	−0.76	0.09	
All	0.11	0.44	−0.33	−0.57	−0.08	
**BMI z-score**						
Gender						0.87
Female	0.09	0.14	−0.05	−0.16	0.06	
Male	−0.06	0.02	−0.08	−0.16	0.00	
Ethnicity						0.61
NZEO	−0.02	0.05	−0.07	−0.16	0.02	
Maori	0.03	0.14	−0.12	−0.28	0.05	
Pacific	0.04	0.06	−0.03	−0.14	0.09	
Baseline VO_2max_						0.89
Low	−0.03	0.00	−0.03	−0.15	0.08	
Medium	0.05	0.12	−0.07	−0.18	0.05	
High	−0.01	0.09	−0.10	−0.24	0.04	
All	0.01	0.09	−0.08	−0.15	−0.01	
**Percentage body fat (%)**						
Gender						0.82
Female	−0.98	0.03	−1.02	−2.39	0.35	
Male	−1.28	−0.53	−0.75	−1.59	0.09	
Ethnicity						0.36
NZEO	−1.49	−1.02	−0.47	−1.36	0.43	
Maori	−0.85	0.02	−0.87	−2.74	1.00	
Pacific	−0.94	0.72	−1.67	−3.19	−0.14	
Baseline VO_2max_						0.47
Low	−1.68	−0.88	−0.80	−1.99	0.38	
Medium	−0.24	−0.05	−0.20	−1.44	1.05	
High	−1.15	0.16	−1.32	−2.67	0.03	
All	−0.99	−0.16	−0.83	−1.54	−0.12	

BMI – body mass index; CI – confidence interval; kg – kilogram; m – metre; NZEO – New Zealand European/Other; VO_2max_ – maximal oxygen uptake.

## Discussion

This exploratory subgroup analysis indicated a consistent intervention effect across subgroups, suggesting active video games could help address weight gain in boys and girls, minority and indigenous groups, and varying levels of fitness. Many health strategies aim to both improve the overall health of the population as well as reduce inequalities within the population. However, these aims are not necessarily achieved in tandem, and interventions that improve overall health may in fact serve to widen the inequality divide via differences in access or uptake [18]. From this perspective, these preliminary findings suggest that an active video games intervention did not appear to perpetuate existing ethnic inequalities in overweight and obesity, as the intervention operated consistently across groups. In addition, in 2005 video game console ownership was slightly higher among Māori (46%) than non-Māori (31%–44%) in New Zealand [19] suggesting that indigenous groups have at least equal access to this type of intervention.

The eGAME trial is the largest study of its type conducted to date, and the sample sizes for the subgroups of interest are larger than many small studies published in the literature. As a standard two-arm RCT, the trial was powered on the primary outcome only, using all randomised participants. The results of all other analyses, including secondary outcomes [13] and mediation [20] reported elsewhere, and the subgroup analyses reported here, are therefore exploratory in the sense that they are considered as hypothesis generating rather than confirmed findings.

*Post hoc* power analyses were not considered appropriate. However, balanced recruitment across ethnicity and gender was sought in order to maximise the statistical power and efficiency of pre-specified subgroup analyses. Despite significant effort, this was not achieved. More than 40% of the sample was Māori or Pacific, but the recruitment target of one third each of Māori, Pacific and New Zealand European/Other was not met. Similarly, more than 70% of participants were male. Despite these imbalances, this study remains one of very few that has specifically targeted minority ethnic groups for participation and reported results separately for these groups [21].

Despite their limitations, subgroup analyses remain a viable approach, and to date provide some of the only available evidence on the effects of interventions on health inequalities. In this study, the difference in treatment effect across subgroups was small, raising issues of cost and feasibility for future trials aiming for sufficient power to detect subgroup differences. Therefore, with appropriately cautious inferences, this study adds to a sparse evidence base.

In conclusion, this active video games intervention operated consistently across important subgroups. These preliminary findings support the use of these games as a pragmatic public health intervention. Researchers interested in pragmatic, community-based interventions should continue to consider the implications of their interventions on health inequalities.

## Abbreviations

BMI: Body mass index; eGAME: The electronic games to aid motivation to exercise study; kg: Kilogram; m: Metre; min: Minute; ml: Millilitre; RCT: Randomised controlled trial.

## Competing interests

The authors declare there are no conflicts of interest. Sony Computer Entertainment Europe (SCEE) provided the gaming software for the study. The study funders (Health Research Council) and SCEE played no role in the design, conduct, or analysis of the study, or in the interpretation and reporting of the study findings.

## Authors’ contributions

The authors’ responsibilities were as follows—RM, CNM, AJ, HP and AR: designed the research (i.e., project conception and development of overall research plan); RM: provided study oversight; LF: conducted the research, undertook data collection and wrote and took primary responsibility for the final content of the manuscript; and YJ: performed statistical analyses. All authors assisted in the interpretation of analyses and revision of the manuscript and read and approved the final manuscript.
